# Supplementary material for: Epigenetic Findings in Twins with Esophageal Atresia
Source: Genes (Basel). 2023 Sep 20;14(9):1822. doi: 10.3390/genes14091822 (PMC10531363; doi:10.3390/genes14091822)
Supplement: Supplementary file 1 [file genes-14-01822-s001.zip › genes-2523905-supplementary.pdf]

Supplementary Table S1. List of hypermethylated and hypomethylated promotors of genes.

Methylation status	Symbols of genes
Hipermethylated promotors of genes	*ABCD1, ACSL4, ADGRG2, AFF2, AHRR, AIFM1, AMER1 AMMECR1, APLN, ARAF, ARHGAP36, ARHGAP4, ARHGAP6, ARHGEF6, ARHGEF9, ARL5C, ATP6AP1, ATP6AP2, BEX2, BEX4, BRCC3, CASK, CCDC160, CD99L2, CHST7, CLCN5, CLIC6, CNKSR2, CSTF2, CUL4B, CXorf58, DACH2, DCAF12L2, DEPTOR, DGKK, DKC1, DUSP22, DUSP9, DYNLT3, ECEL1P2, EFNB1, EMD, ESX1, FAM104B, FAM120C, FAM156A, FAM199X, FAM3A, FGD1, FGF13, FHL1 FMR1, FRMPD4, FUNDC2, GABRE, GABRQ, GDI1 GDNF, GK, GLUD2, GPC3, GPC4, GPR101, GPR50, GPRASP1, GPRASP2, GRIA3, GSPT2, HCCS, HMGB3, HPRT1, HS6ST2, HTATSF1, IDS, IGBP1, IKBKG, INGX, IRAK1, IRS4, JADE3, KCNE5, KLF8, KLHL13, KLHL34, KRBOX4, LAS1L, LINC00629, LINC02226, LOC729609, LONRF3, MAGED2, MAGEE1, MAMLD1, MAOA, MAP3K15, MAP7D2, MAP7D3, MBNL3, MCF2, MED12, MID1, MID1IP1, MID2, MIR572, MOSPD1, MPP1, MSL3, MSN, MTM1, MTMR8, NDUFA1, NEXMIF, NHS, NHSL2, NKAP, NKAPP1, NLGN2, NR0B1, NR4A2, NUDT10, OCRL, OTUD5, PAK3, PCDH19, PDHA1, PDK3, PDZD4, PGK1, PGRMC1, PHF8, PIGA, PJA1, PLXNA3, PNCK, POLA1, POU3F4, PPP1R3F, PQBP1, PRAF2, PRPS1, PRPS2, PRRG3, RAB33A, RAB39B, RAD9B, RAI2, RBBP7, RBM46, RLIM, RP2, RPGR, RPL10, RPS6KA3, RPS6KA6, RTL5, RTL8C, SAT1, SCART1, SCML2, SH2D4B, SH3KBP1, SHROOM4, SLC10A3, SLC16A2, SLC25A14, SLC25A43, SLC25A53, SLC9A6, SLC9A7, SLITRK2, SMIM10L2A, SMIM10L2B, SNX12, SOWAHD, SOX3 SRPX, SSR4, STAG2, STARD8, SUV39H1, SYP, SYTL4, TAB3, TAF1, THOC2, TMEM164, TMEM47, TMSB15A, TSC22D3, TSPAN6, TSPAN7, UBA1, UBE2A, UBQLN2, USP27X, USP51, UXT-AS1, VMA21, WDR13, WDR44, WDR45, WNK3, XIAP, XKRX, YIPF6, ZBTB33, ZC4H2, ZCCHC12, ZCCHC18, ZDHHC9, ZIC3, ZMYM3, ZNF185, ZNF275, ZNF280C, ZNF41, ZNF449, ZNF630, ZXDB,*
Hypomethylated promotors of genes	*A2M-AS1, AATK, ACAP3, ADGRG6, ALOX12, ARHGAP45, ARL6IP1, B4GALT2, BRSK2, CCDC43, CD70, CILP2, CIT, COASY, CSRNP1, DOK7, DUS3L, DYRK4, EBF1, EHMT1, FBXO46, GLE1 HDAC7, HTRA4, IVD, KCNJ12, KLF6, LARGE2, LDLRAD4, LHX6, LINC00963, MAP3K14-AS1, MATK, MCAM, MEGF9, MEIS3P1, MIR196A1, MIR3074, MIR3143, MIR4648, MIRLET7BHG, MSH6, NCOR2, NQO2, OR2L13, OSMR, OXLD1, PEBP1, PER3, PKP3 PLCB2, PLEKHG6, PML, PRR34-AS1, PTPRU, PYCR1, RILP, RUNX3, S100A11, SELENOO, SH2D3C, SHANK3, SMG1P2, STK19, TFR2, TIFA, TNFAIP2, TONSL, TRIM29, TRIP6, TXNDC5, URGCP, VGLL4, ZDHHC4, ZNF177, ZNF441, ZNF766, ZNF786,*

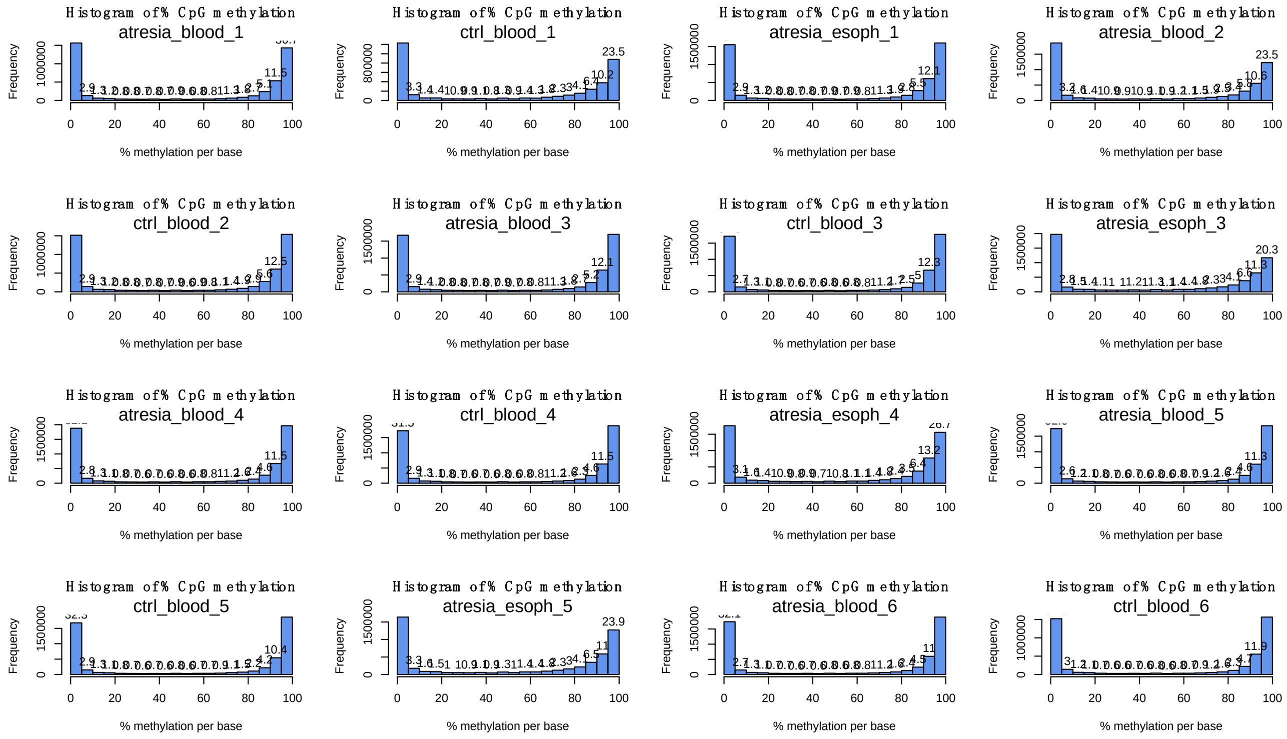

Supplementary Figure S1. Histograms illustrating the distribution of methylation levels in all examined samples. X-axis represents the frequency of methylation, while the Y-axis represents the occurrence frequency. A proper chart for a sample should exhibit two peaks, one on the left side (0%) and the other on the right side (100%), reflecting the characteristic methylation pattern of cytosines, where either no methylation (0%) or complete methylation (100%) is most commonly observed. Histograms for all samples met this criterion.

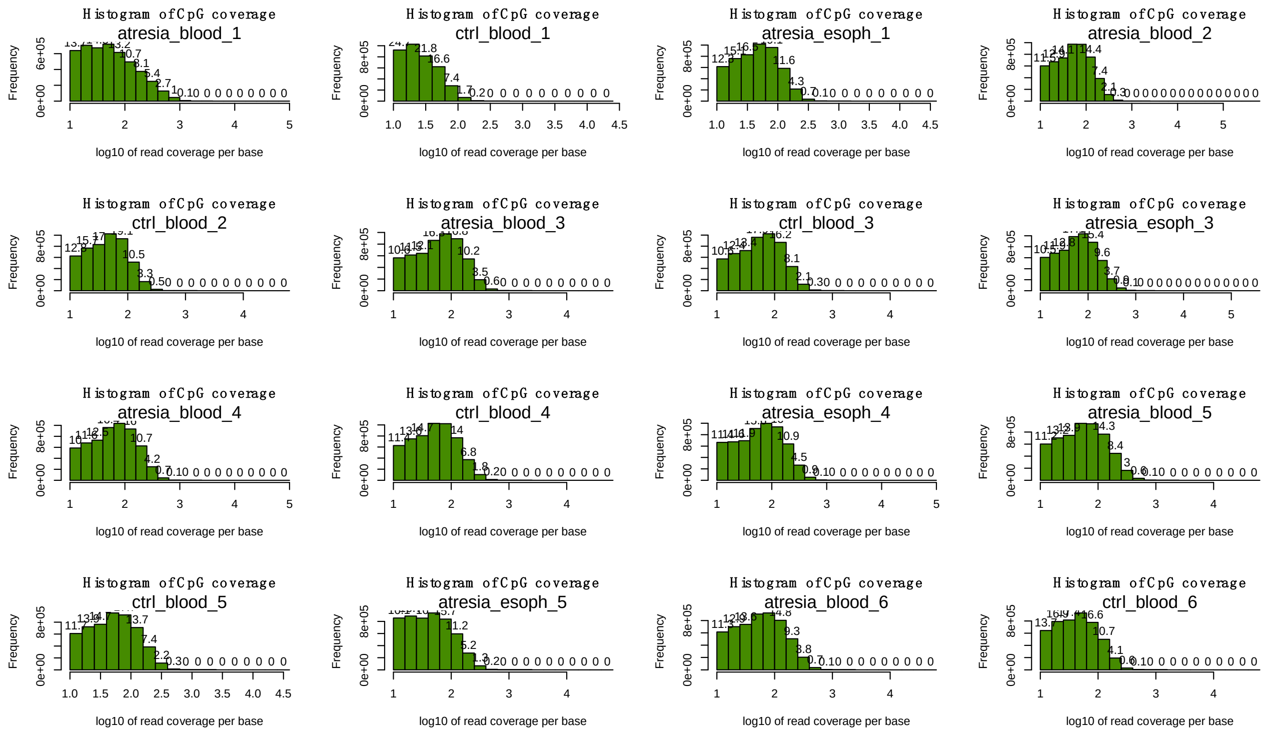

Supplementary Figure S2. Histograms illustrating the coverage in all examined samples. The X-axis represents the logarithmically scaled coverage

(e.g., 10 reads per nucleotide on this scale equals 1), while the Y-axis represents the occurrence frequency. A proper histogram should decline from left to right. The presence of abnormal duplications (during the PCR stage) or preferential amplifications would result in an additional peak appearing on the right side of the histograms. No abnormalities were detected in any of the examined samples.
